# Abnormal Expression of ERα in Cholangiocytes of Patients With Primary Biliary Cholangitis Mediated Intrahepatic Bile Duct Inflammation

**DOI:** 10.3389/fimmu.2019.02815

**Published:** 2019-12-05

**Authors:** Hui Cao, Bukun Zhu, Yao Qu, Wei Zhang

**Affiliations:** Department of Liver Diseases, Longhua Hospital, Shanghai University of Traditional Chinese Medicine, Shanghai, China

**Keywords:** ERα, pro-inflammatory cytokines, MAPKs, inflammatory cellular phenotype, PBC

## Abstract

ERα, one of the classical receptors of estrogen, has been found to be abnormally up-regulated in patients with primary biliary cholangitis (PBC), which is an important factor leading to ductopenia. ERα-mediated signaling pathways are involved in proliferation of human intrahepatic biliary epithelial cells (HiBECs) and portal inflammation. Our previous studies have shown that the expression levels of ERα in the liver tissues of PBC patients are positively correlated with the levels of serum pro-inflammatory cytokines. The present study was designed to assess the relationship between abnormal ERα expression in small bile ducts and the progression of PBC. We examined the levels of multiple cytokines and analyzed their relationship with clinical parameters of livers functions in a cohort of 43 PBC patients and 45 healthy controls (HC). The levels of ERα expression and the relation with the levels of cytokines were further assessed. The localization of cytokines and ERα-mediated signaling pathways in liver were examined using immunohistochemistry. The possible underlying mechanisms of these alterations in PBC were explored *in vitro*. Our results demonstrated that the levels of IL-6, IL-8, and TNF-α were increased in PBC patients, and positively correlated with the serum AKP levels and ERα expression levels. Moreover, the expression of these cytokines were up-regulated in HiBECs that were stimulated with 17β-estradiol and PPT (an ERα agonist) and they also were positive in intrahepatic bile duct of PBC patients. The ERα-mediated expression of pro-inflammatory cytokines was induced by JNK, P38, and STAT3 phosphorylation in HiBECs. In addition, the CD54 expression was increased in HiBECs after ERα activation, which induced peripheral blood monouclear cells (PBMCs) recruitment. In conclusion, the present study highlighted a key role of abnormal ERα expression in inducing an inflammatory phenotype of HiBECs, which was critical in the development of inflammation and damage in small bile duct.

## Introduction

Primary biliary cholangitis (PBC) is an occult and chronic progressive autoimmune liver disease, and, if untreated, will culminate into end-stage biliary cirrhosis, leading to liver failure (1). Women are more susceptible to suffering this disease than men, although some recent data suggest an increasing male prevalence (2). According to epidemiologic studies, 1 in 1,000 women over the age of 40 live with PBC, and the estimated incidence is between 1 and 2 per 100,000 population per year in European populations (3); commonly cited ranges for incidence and prevalence are 0.3–5.8 and 1.9–40.2 per 100,000, respectively (4). Nevertheless, the female predominance of PBC continues to be unexplained. The clinical features of PBC are persistent intrahepatic cholestasis and serologic reactivity to anti-mitochondrial antibodies (AMA) or specific antinuclear antibody (ANA) reactivity, with the pathological features being chronic non-suppurative, granulomatous, lymphocytic small bile duct cholangitis (5). The onset of the cholangitis is related to the multiple immune cells infiltration in the surrounding of the small bile duct. Previously, Tsuneyama et al. have reviewed the types of immune cells which infiltrate into the peripheral of small bile ducts in PBC patients; they have found that T cells comprise 55% of the cellular infiltrate, macrophages and B cells/plasma cells account for ~30 and 10% in early stage, respectively, showing chronic non-suppurative destructive cholangitis (CNSDC), and these activated immune cells play important roles in initiating the breakdown of tolerance (6). Therefore, some scholars believe that the pathological starting point of PBC is autoimmune-mediated injury of small bile duct, and the immunological interaction between human intrahepatic biliary epithelial cells (HiBECs) and surrounding inflammatory cells is a pivotal mechanism to trigger small bile duct lesions (7). Interestingly, HiBECs, which are the main damaged cells in the early stage of PBC, may not be simply considered as an “innocent victim” of immune attack (8)_._ Cholangiocytes in small bile duct of PBC patients are expressing various cytokines and chemokines in order to generate and sustain the specific surrounding inflammatory conditions, which will induce HiBECs apoptosis and exacerbate bile duct injury (6). Making the matters worse, the damaged HiBECs with aberrant expression of human leukocyte antigen (HLA) class II and other co-stimulatory molecules have an antigen-presenting ability (9). Hence, immunogenicity of HiBECs will be amplified. In addition, HiBECs in damaged biliary duct will have up-regulated expression of phagocytosis related receptor phosphatidylserine receptor (PSR), which is involved in phagocytosis of adjacent injured and apoptotic peers due to immunological interaction (10). And, normal HiBECs will express pyruvate dehydrogenase complex E2 (PDC-E2) after phagocytosed the adjacent injured and apoptotic peers (11). As a result, the immune tolerance of HiBECs will be impaired. Therefore, various types of migratory inflammatory cells become effector cells that are more aggressive to HiBECs (11, 12). However, effector cells also generate additional cytokines and chemokines to produce an inflammatory status and progressive fibrosis (12). Unfortunately, there is no clear mechanism to explain the causes of the onset abnormal activation of HiBECs.

Based on the clinical features of PBC that is predominant among perimenopause women, a previous study has shown that the abnormal expression of estrogen receptor α (ERα) in HiBECs of PBC patients is an important reason leading ductopenia (13). ERα is one of the classical receptors of estrogen, and is often expressed at high levels in uterus, ovary, pituitary gland, vas deferens, and adipose tissues (14). Apparently, liver is also the target organ of estrogen, and the intrahepatic bile duct has up-regulated expression of ERα under certain conditions (partial hepatectomy and bile duct ligation). However, ERα-mediated signaling pathway can play multiple roles in bile duct, which stimulates HiBECs proliferation (15, 16) and is involved in portal inflammation that was induced by environmental xenoestrogens (17). Recently, some researchers have found that the soil in the high incidence area of PBC contains a large amount of xenobiotic that can activate ERα (18). However, this study does not fully elucidate the relationship between ERα activation and pathogenesis of PBC.

In a preliminary study on the relationship of expression levels of ERα and serum cytokines of PBC patients, we found the expression levels of ERα in liver of PBC patients were positively correlated with the serum levels of pro-inflammatory cytokines, such as IL-6, IL-8, and TNF-α. In addition, it was further confirmed that ERα was mainly expressed in the intrahepatic bile duct of the PBC patients. Therefore, we hypothesized that HiBECs would transform into an “inflammatory cellular phenotype” (higher expression pro-inflammatory cytokines and adhesion molecule) following ERα activation resulting in multiple immune cells being recruited, and immune homeostasis of small bile duct being disturbed. The immunologic injury of HiBECs would evoke the onset of PBC. The present study was designed to test the above hypothesis *in vitro* and *in vivo*.

## Materials and Methods

### Patient Characteristics and Liver Tissue Samples

Blood samples were collected from 43 PBC patients, before any ursodeoxycholic acid (UCDA), corticosteroids and sex hormone therapy at the Longhua hospital of Shanghai University of Traditional Chinese Medicine. Blood samples were also collected from 45 volunteers as healthy controls, who had normal ranges of routine physical examination (fasting blood glucose, liver and kidney function tests, blood lipids, urine, and stools) and without any acute or chronic liver diseases during routine check-up at the same hospital. The 10-mL peripheral blood sample was collected from all the members and the concentrations of cytokines were measured. The liver biopsies were obtained from 8 post-menopausal women who needed to confirm this diagnosis by liver pathology. The pathological results confirmed the diagnoses of PBC in the 8 patients: 4 at stage II, 3 at stage III, and 1 at stage IV. As controls, 5 liver biopsies all showed normal histology. All PBC patients were negative for hepatitis B and C markers.

### Cell Culture and Stimulation

HiBECs were purchased from tongpai biotechnology co., Ltd, and the cell line was confirmed by the expression of the biliary-type cytokeratins CK19 (using immune fluorescence) (Supplementary Figure 1A) and CK7 (using immunoblotting) (Supplementary Figure 1D), and the morphology of HiBECs was observed by optical microscope (Supplementary Figures 1B,C). HiBECs were suspended in RPMI 1640 medium (Invitrogen, California, USA) with 10% fetal bovine serum supplemented with 10 ng/mL of epidermal growth factor (Sigma-Aldrich, Munich, Germany), 5 mg/mL insulin (Sigma-Aldrich, Munich, Germany), 100 units/ml penicillin, and 100 mg/ml streptomycin (Invitrogen, California, USA). When cells reached 60–70% of confluence, 17β-estradiol (Sigma-Aldrich, Munich, Germany), ERα agonist 4,4,4-(4-propyl-[1H] pyrazole-1,3,5-triyl)-tris-phenol (PPT) (Sigma-Alrich, Munich, Germany), ERβ agonist 2,3-bis (4-hydroxyphenyl)-propionitrile (DPN) (Sigma-Aldrich, Munich, Germany) and ERα antagonist fulvestrant (ICI182, 780) (Sigma-Aldrich, Munich, Germany) at various concentrations were added into the medium. The cells were cultured for 48 h and then harvested for further PCR and immunoblotting analyses.

### Real-Time PCR Analysis

Real-time PCR analysis was performed using the SYBR-Green mix (Applied Biosystems, Foster City, CA, USA) in an ABI PRISM 7500HT sequence detector. Primers used for real-time RTPCR are shown in Supplementary Table 2. Briefly, total RNA was isolated and 1 mg of RNA was reverse transcribed using RNA reverse transcription Kit (Takara, Kusatsu, Japan). The resultant cDNA was appropriately diluted and amplified in the SYBR-Green mix 10-ml system. The mRNA expression levels of IL-6, IL-8, TNF-α, and other cytokines were normalized to the level of β-actin mRNA.

### Immunoblotting Analysis

HiBECs were first lysed in RIPA buffer for 30 min. The samples were separated by 12% SDS polyacrylamide gel electrophoresis (SDS-PAGE) at 80 V for 0.5 h and 100 V for 1 h before transfer onto polyvinylidene difluoride membranes (PVDF). After blocking, the blots were incubated overnight with 4°C with hybridized with primary Abs. ERα (1:200) (Novus Biologicals, Colorado, USA), ERβ (1:500) (R&D Systems, Minnesota, USA), P-JNK (1:250) (CST, Boston, USA), P38 (1:500) (CST, Boston, USA), P-STAT3 (1:200) (CST, Boston, USA), and GAPDH (1:1000) (CST, Boston, USA). The blots were then washed with PBST and then incubated for 1 h with secondary antibodies conjugated to horseradish peroxidase (1:5000) (CST, Boston, USA). The membranes were scanned with a LAS-4000 luminescent image analyzer (Bio-Rad, California, USA). Digital images of resultant chemiluminescent signals were analyzed using Image Lab software (Bio-Rad, California, USA).

### PBMC Preparation and Adhesion Assay

Freshly isolated peripheral blood mononuclear cells (PBMCs) were extracted from 20 mL of fresh heparinized blood samples of PBC patients and healthy controls, using human lymphocyte separation solution (Sigma-Aldrich, Munich, Germany). The PBMCs were cultured in RPMI 1640 medium supplemented with 10% fetal bovine serum, 100 mg/mL of penicillin, and 100 mg/mL of streptomycin. To obtain mitogen- activated lymphocytes, the PBMCs were stimulated with 10 mg/mL of phytohemag glutinin (Sigma-Aldrich, Munich, Germany) for 72 h at 37°C. The cell adhesion assay was modified as described previously (19). Briefly, the PBMCs were labeled with BCECF-AM (5 μg/mL) (Beyotime Biotechnology, Shanghai, China) for 1 h at 37°C, washed three times with sterile PBS, and re-suspended in serum free RPMI 1640 medium. The HiBECs were incubated with reagents on a 24-well culture plate, and then co-cultured with BCECF-AM-labeled PBMCs for 1 h at 37°C. Non-adhering PBMCs were removed by pipette, and the wells were washed with sterile PBS. The PBMCs binding to HiBECs were measured by fluorescence microscope. The fluorescence intensity was analyzed by ImageJ (National Institutes of Health, Bethesda, MD, USA).

### Flow Cytometric Analysis

Flow cytometry was performed as described previously (20). Briefly, HiBECs were incubated with reagents on a 6-well culture plate for 48 h, and then rapidly digested and centrifuged, washed, re-suspended and stained according to standard protocols using the following mAbs:CD54 (BD Biosciences, New Jersey, USA), CD106 (BD Biosciences, New Jersey, USA), and CD58 (BD Biosciences, New Jersey, USA). These markers were used to observe the changes of adhesion molecules in HiBECs after incubated with reagents. Data acquisition was performed using a BD Biosciences LSR Fortessa cytometer (BD Biosciences, New Jersey, USA), and results were analyzed using FlowJo analysis software (BD Biosciences, New Jersey, USA).

### Immunofluorescence Analysis

The protocol was performed as our laboratory standard. Briefly, formalin-fixed paraffin-embedded tissue sections were deparaffinized and rehydrated. Blocking of endogenous avidin/biotin, and antigen retrieval was performed by boiling the sections in 0.01 M sodium citrate for 24 min. The sections were then incubated in 0.1% triton for 10 min and blocked with 5% BSA for 60 min, and then incubated with primary antibodies: rabbit anti-human CD54 antibody (CST, Boston, USA), and mouse anti-human ERα antibody (Novus Biologicals, Colorado, USA), at 4°C overnight. Following washing, the tissues sections were incubated with alexa fluor 594-affinipure donkey anti-mouse IgG (Yeasen, Shanghai, China) and alexa fluor 488-affinipure goat anti- rabbit IgG (Yeasen, Shanghai, China) for 60 min at 37°C. Finally, the cell nuclei were stained by DAPI. The results were examined under a fluorescence microscope.

### Immunohistochemistry

Immunohistochemistry analysis was completed as previously reported (21). Briefly, formalin-fixed paraffin-embedded tissue sections were deparaffinized and rehydrated. Blocking of endogenous avidin/biotin, and antigen retrieval was performed by boiling in 0.01 M sodium citrate for 24 min. The sections were then blocked with 5% BSA for 30 min, and then incubated with primary antibodies: rabbit anti-human P-JNK antibody (CST, Boston, USA), rabbit anti-human P-STAT3 antibody (CST, Boston, USA), mouse anti-human ERα antibody (Novus Biologicals, Colorado, USA), mouse anti-human ERβ antibody (R&D Systems, Minnesota, USA), rabbit anti-human IL-6 antibody (CST, Boston, USA), mouse anti-human IL-8 antibody (Santa Cruz Biotechnology, Dallas, USA), rabbit anti-human TNF-α antibody (CST, Boston, USA), rabbit and anti-human CK-19 antibody (HUA BIO, Hangzhou, China) at 4°C overnight. Following washing, tissues sections were incubated with horseradish peroxidase conjugated anti-mouse or rabbit anti-bodies (CST, Boston, USA) for 30 min at 37°C. Specific staining was detected by 3, 3-diaminobenzidine and examined by light microscopy. Tissue sections were evaluated using Image pro plus (Media cybernetics, Maryland, USA).

### Statistical Analysis

The statistical significance of the differences in the levels of cytokines between PBC patients and healthy controls were analyzed using Non-parametric test. Linear relation were performed using Spearman's rank correlation to assess the relationship between ERα expression levels, cytokine levels and liver function. Independent samples were analyzed using Student's *t*-test or one-way analysis of variance unless indicated in the figure legends. Statistical analyses were performed on raw data using Graph Pad software and SPSS 21. All of the *P*-values were shown as two-sided; and *P* < 0.05 was considered statistically significant.

## Results

### The Serum Levels of IL-6, IL-8, and TNF-α in PBC Patients Are Higher Than That in Control Subjects

**Figure 1:**
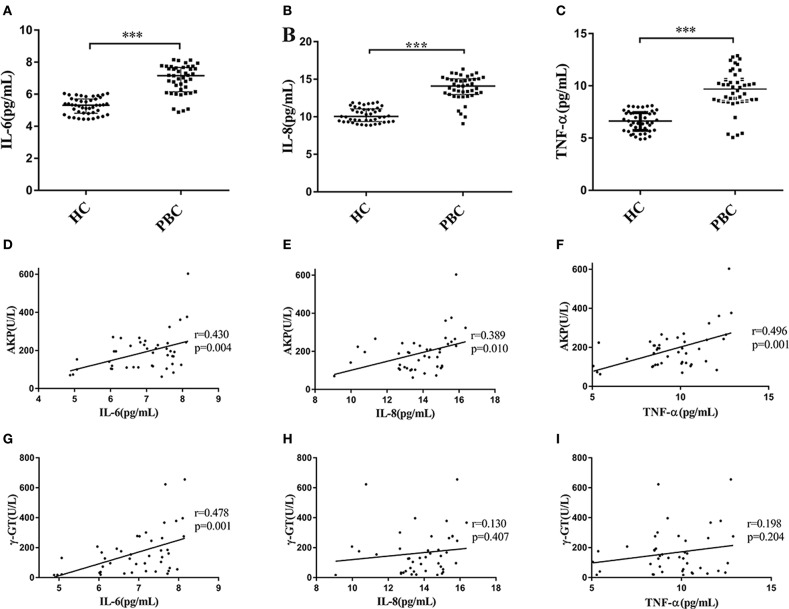
Serum levels of cytokines and correlation with AKP and γ-GT. **(A–C)** Serum levels of IL-6 **(A)**, IL-8 **(B)**, and TNF-α **(C)** in PBC patients (*n* = 43) and HC (*n* = 45), ***P < 0.001, compared with HC by a Mann–Whitney test. Data are represented as median with interquartile range. **(D–F)** Serum IL-6 **(D)**, IL-8 **(E)**, and TNF-α **(F)** concentration with the levels of AKP in PBC patients (*n* = 43). *r* = 0.430, *p* = 0.004 (IL-6); *r* = 0.389, *p* = 0.010 (IL-8) and *r* = 0.496, *p* = 0.001 (TNF-α), respectively. **(G–I)** Serum IL-6 **(G)**, IL-8 **(H)**, and TNF-α **(I)** concentration with the levels of γ-GT in PBC patients (*n* = 43). *r* = 0.478, *p* = 0.001 (IL-6); *r* = 0.130, *p* = 0.407 (IL-8) and *r* = 0.198, *p* = 0.204 (TNF-α), respectively. The *p*-values were determined by Spearman's rank correlation, *P* < 0.05 was considered significant. PBC, primary biliary cholangitis; HC: healthy control; IL-6, interleukin-6; IL-8, interleukin-8; TNF-α; tumor necrosis factor-alpha; AKP, Alkaline phosphatase; γ-GT, g-glutamyl transpeptidase.

We recruited 43 PBC patients and 45 healthy controls (HC) in the present study. The mean ages of PBC patients and HC were almost the same, and the characteristics of PBC patients and healthy controls are shown in Supplementary Table 1). The median values of AKP, γ-GT and TBiL in PBC patients were 195.29 U/L, 131.00 U/L and 13.20 μmol/L, respectively. We next tested the serum multiple cytokines levels of PBC patients and HC (Supplementary Figure 2). We found that the levels of IL-6, IL-8, and TNF-α in the patients were higher than that in HC (Mann-Whitney test *Z* = −6.997, *P* < 0.001; *Z* = −7.180, *P* < 0.001; *Z* = −6.599, *P* < 0.001, respectively) (Figures 1A–C). Moreover, as Li et al. (22) proposed, the serum abnormal cytokines levels may have a linear relationship with the parameters for liver functions (AKP, γ-GT, and TBiL) in PBC patients. As expected, the results from the present study showed that there were a positive correlations between the levels of AKP and IL-6 (*r* = 0.430, *p* = 0.004), IL-8 (*r* = 0.389, *p* = 0.010) and TNF-α (*r* = 0.496, *p* = 0.001) (Figures 1D–F). However, the levels of γ-GT were not positive correlated with IL-8 (*r* = 0.130, *p* = 0.407), and TNF-α (*r* = 0.198, *p* = 0.204), except for IL-6 (*r* = 0.478, *p* = 0.001) (Figures 1G–I). In addition, we did not find the levels of TBiL has linear relationships with the serum levels of IL-6, IL-8, and TNF-α Supplementary Figures 2M–O). And there were no correlations with other cytokines (Supplementary Figures 2A–S).

### The ERα Expression Levels in Small Bile Ducts Are Positively Correlated With the Serum Cytokines Levels in PBC Patients

**Figure 2:**
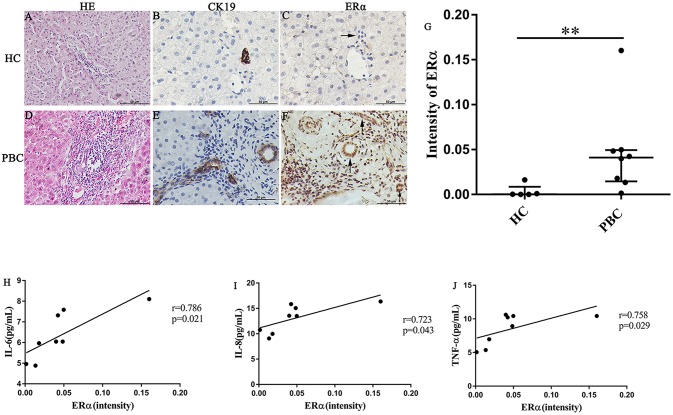
ERα expression in liver biopsies of PBC patients, HC and correlation with cytokines. **(A–J)** A significant immunohistochemical ERα positive expression in cholangiocytes of PBC patients **(F)** (*n* = 8, both cytoplasm and nucleus) (lower right panel), but negatively in HC (*n* = 5) (upper right panel) (original magnification, ×400). **(G)** ERα expression was higher in patients with PBC, **p < 0.01, compared with HC by a Mann–Whitney test. Data are represented as median with interquartile range. **(H–J)** Serum IL-6 **(H)**, IL-8 **(I)**, and TNF-α **(J)** level with the intensity of ERα in PBC patients. The *p*-values were determined by Spearman's rank correlation, *P* < 0.05 was considered significant. PBC, primary biliary cholangitis; HC: healthy control; IL-6, interleukin-6; IL-8, interleukin-8; TNF-α; tumor necrosis factor-alpha; ERα, estrogen receptor alpha.

In our previous study, we found that the ERα expression levels in the liver were positively correlated with the concentrations of various pro-inflammatory cytokines in PBC patients. However, the location of ERα expression was not detected precisely. Hence, we selected some liver biopsies from 8 PBC patients, the location and levels of ERα expression in the small bile ducts were tested by immunohistochemistry analysis. Then, we assessed the linear relationship between ERα expression levels and serum cytokines levels. In addition, to find out the small bile ducts in portal area, we selected biliary-type cytokeratin CK19 as a biomarker and determined the ERα expression level in the CK19-positive areas. The results of immunohistochemistry showed that positive expression of ERα (both cytoplasm and nucleus) was mainly located in intrahepatic bile ducts, which was consistent with CK19-positive area (lower right panel) (Figures 2E,F). Similar to previous research, normal small bile ducts in HC did not express ER (upper right panel) (Figures 2B,C). Further analysis indicated that ERα expression levels in small bile duct of PBC patients were significantly higher than that in HC (Figure 2G).

Next, the linear relationship between ERα expression levels and serum levels of IL-6, IL-8 and TNF-α was analyzed using spearman's rank correlation. Indeed, there were a positive correlations between ERα and the cytokines (*r* = 0.786, *p* = 0.021; *r* = 0.723, *p* = 0.043 and *r* = 0.758, *p* = 0.029 for IL-6, IL-8, and TNF-α, respectively) (Figures 2H–J). Although ERβ expression in small bile ducts of PBC patients was detected by immunohistochemistry, the ERβ expression levels were not related with cytokines levels (Supplementary Figure 3).

### 17β-Estradiol Induces the Expressions of IL-6, IL-8, and TNF-α in HiBECs

**Figure 3:**
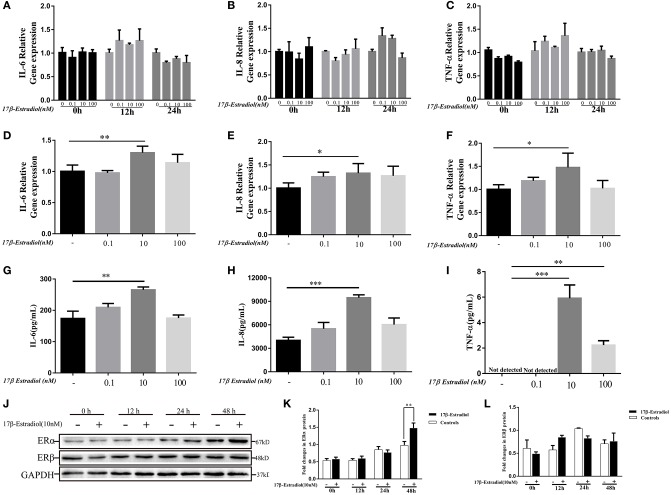
*In vitro* 17β-estradiol induces cytokines secretion in HiBECs. HiBECs were stimulated with 3 different concentrations of 17β-estradiol (0.1, 10, and 100 nM) for four time-points (0, 12, 24, and 48 h). Total RNA was isolated and analyzed by real-time PCR for IL-6 mRNA, IL-8mRNA and TNF-α mRNA expression. **(A–C)** The resulted shown that the levels of IL-6mRNA **(A)**, IL-8mRNA **(B)**, and TNF-α mRNA **(C)** were not changed, when compared with controls at 0, 12, and 24 h. **(D–F)** The expression of IL-6mRNA **(D)**, IL-8mRNA **(E)**, and TNF-α mRNA **(F)** were up-regulated at 48 h. And, 10 nM 17β-estradiol can up-regulate expression of IL-6mRNA, IL-8 mRNA, and TNF-α mRNA. *p < 0.05, **p < 0.01, compared with controls by Student *t*-test. **(G–I)** Elisa analysis of IL-6, IL-8 and TNF-α protein in cellular supernatants from 17β-estradiol-induced HiBECs at 48 h. **p < 0.01, ***p < 0.001, compared with controls by Student *t*-test. **(J)** Immunoblotting analysis of ERα and ERβ expression in HiBECs that were stimulated with 17β-estradiol (10 nM) at four time points (0, 12, 24, and 48 h). **(K)** 17β-estradiol (10 nM) had no effect on ERα expression at 0, 12, and 24 h, but up-regulated ERα expression at 48 h, **p < 0.01 compared with controls by Student *t*-test. **(L)** 17β-estradiol (10 nM) had no effect on ERβ expression at four time points. All data are representative of three independent experiments.

As shown in Figure 2, the expression levels of ER α were involved in pro-inflammatory cytokines expression in PBC patients. Hence, we wanted to confirm it *in vitro*. 17β-estradiol was a classic steroid hormone was selected and its concentrations used *in vitro* were roughly equivalent to those in postmenopausal female (0.1 nM), normal premenopausal female (10 nM), and pregnant female (100 nM) (21), respectively. Under normal conditions, HiBECs did not or slightly expressed ERα protein. In the present study, we observed the ERα and cytokines (IL-6, IL-8, and TNF-α) expressions in HiBECs that were stimulated by three concentrations of 17β-estradiol at four time-points (0, 12, 24, and 48 h). The RT-PCR results showed that the expression levels of IL-6, IL-8, and TNF-α mRNAs were not changed in HiBECs that were stimulated with 17β-estradiol for 12 and 24 h (Figures 3A–C). However, the expression levels of IL-6, IL-8, and TNF-α mRNA in HiBECs that was treated with 10 nM 17β-estradiol for 48 h were higher than that in the controls (Figures 3D–F). It is worth noting that higher doses of 17β-estradiol might have a negative impact on cytokines expression [the immunosuppressive action of high concentration of estrogen (12)]. Subsequently, the levels of cytokines (IL-6, IL-8, and TNF-α) protein in the cell culture supernatant were analyzed by Elisa kit. The results were consistent with RT-PCR, and 10 nM 17β-estradiol might be an ideal reagent to induce expression of IL-6, IL-8, and TNF-α in HiBECs (Figures 3G–I). Finally, we detected the expression of two kinds of estrogen receptors (ERα and ERβ). HiBECs were stimulated with 17β-estradiol (10 nM) for four time periods (0, 12, 24, and 48 h), and ERα and ERβ proteins were analyzed by immunoblotting. We found the levels of ERα expression were gradually increased along with the increase in exposure time, but did not find the statistic difference among control groups and 17β-estradiol groups at 0, 12, and 24 h (Figures 3J,K). However, the levels of ERα expression in HiBECs at 48 h were significantly higher than that in control groups (Figures 3J,K). And, it was not observed any significant difference in ERβ expression between control groups and 17β-estradiol groups (Figures 3J,L). Taken together, these data provided an evidence that 17β-estradiol could induce cytokines expression in HiBECs via activated ERα.

### Fulvestrant Effectively Blocks 17β-Estradiol-Mediated Cytokines Expression in HiBECs

In order to further verify that the 17β-estradiol-mediated pro-inflammatory cytokines expression in HiBECs was dependent of ERα activation. we used the ERα agonist 4,4,4-(4-propyl-[1H] pyrazole-1,3,5-triyl)-tris-phenol (PPT) and the ERβ agonist 2,3-bis (4-hy droxyphenyl)-propionitrile (DPN) that were added to HiBECs parallel with 17β-estradiol (10 nM) or vehicle in the present experiments. And the fulvestrant (ICI182, 780, ERα antagonist) was added to HiBECs 2 h before stimulation with 17β-estradiol.

**Figure 4:**
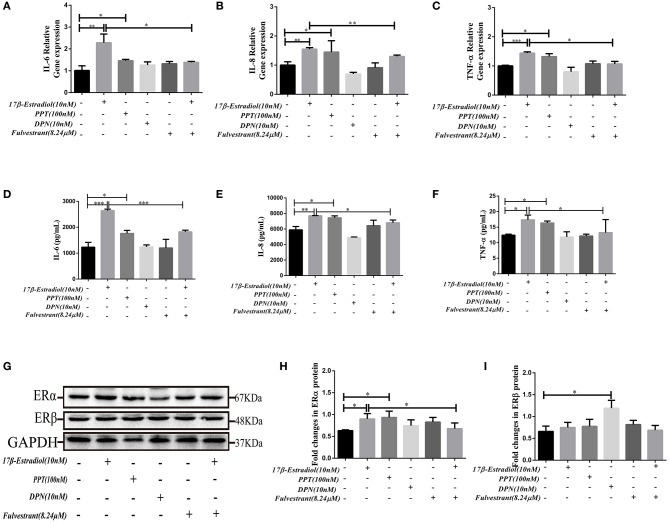
*In vitro* fulvestrant inhibits ERα expression and cytokines secretion. **(A–C)** PPT (100 nM), DPN (10 nM) were added to HiBECs parallel with 17β-estradiol (10 nM) or vehicle. And, fulvestrant (8.24 μM) was added to HiBEC 2 h before stimulation with 17β-estradiol (10 nM). Total RNA was isolated and analyzed by real-time PCR for IL-6mRNA, IL-8mRNA, and TNF-α mRNA expression. The resulted showed that PPT treatment significantly induced IL-6 mRNA **(A)**, IL-8mRNA **(B)**, and TNF-α mRNA **(C)** expression as well as 17β-estradiol. In contrast, DPN had no obvious effects on them. In addition, pretreatment with fulvestrant (8.24 μM) inhibited IL-6mRNA, IL-8mRNA, and TNF-α mRNA expression. *p < 0.05, **p < 0.01, ***p < 0.001, compared with controls or 17β-estradiol group by one-way analysis of variance. **(D–F)** ELISA analysis of IL-6 **(D)**, IL-8 **(E)**, and TNF-α **(F)** protein in cell culture supernatants. The results were consistent with RT-RCR *p < 0.05, **p < 0.01, ***p < 0.001, compared with controls or 17β-estradiol groups by one-way analysis of variance. **(G–H)** Immunoblotting analysis of ERα and ERβ expression levels in HiBECs that were stimulated with PPT, DPN, fulvestrant, and 17β-estradiol. **(H)** The expression of ERα in HiBECs was up-regulated by 17β-estradiol and PPT, and fulvestrant effectively blocked ERα expression (*n* = 3, mean ± SEM). *p < 0.05, determined by ANOVA with a Tukey test. **(I)** ERβ expression was not changed in all groups, except for the DPN groups. *p < 0.05, compared with controls. All data are representative of three independent experiments.

The results showed that PPT (100 nM) could up-regulate the IL-6, IL-8, and TNF-α mRNA expression in HiBECs (Figures 4A–C), and it was similar to 17β-estradiol (10 nM). However, we did not observed that the expression of IL-6, IL-8, and TNF-αmRNA in HiBECs were up-regulated by stimulation of DPN (Figures 4A–C). As expected, the IL-6, IL-8, and TNF-α mRNA expressions in HiBECs were significantly down-regulated by pretreatment with fulvestrant (8.24 μM) for 2 h (Figures 4A–C). Then, the cytokines proteins in the cell culture supernatant of all groups were analyzed by Elisa kits. The results were consistent with RT-PCR, and the protein levels of IL-6, IL-8, and TNF-α in HiBECs that were treated with 17β-estradiol (10 nM) and PPT (100 nM) were higher than that in controls (Figures 4D–F). Indeed, fulvestrant (8.24 μM) blocked 17β-estradiol-mediated these cytokines expression in HiBECs (Figures 4D–F).

Finally, the ERα expression levels in HiBECs that were stimulated with PPT, DPN, 17β-estradiol, and fulvestrant were tested by immunoblotting analyses. The results showed that the ERα expression levels were up-regulated in HiBECs that were treated with 17β-estradiol and PPT (Figures 4G,H), and fulvestrant showed an inhibitory effect on ERα expression (Figures 4G,H). However, ERβ expression was not changed in all groups, except for the DPN groups (Figures 4G,I). Taken together, these data provided additional evidences for that ERα activation was accounting for pro-inflammatory cytokines expression in HiBECs.

### The ERα-Mediated IL-6, IL-8, and TNF-α Expressions in HiBECs Are via Activated MAPKs Pathway

**Figure 5:**
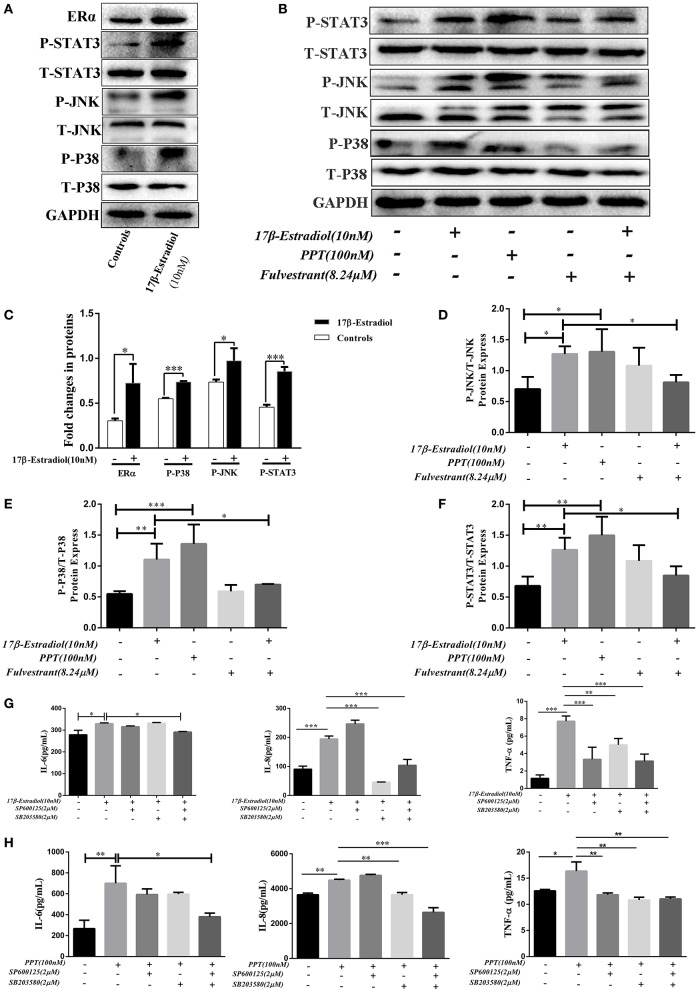
ERα-mediated JNK, P38 and STAT3 activation. **(A)** Immunoblotting analysis of JNK, P38, and STAT3 activation in HiBECs which were stimulated with 17β-estradiol (10 nM). **(B)** Immuno blotting analysis of JNK, p38, and STAT3 activation in HiBECs which were stimulated with PPT (100 nM), fulvestrant (8.24 μM), and 17β-estradiol (10 nM). **(C)** Significant increase of JNK, P38, and STAT3 phosphorylation following 17β-estradiol treatment (*n* = 3, mean ± SEM). *p < 0.05, ***p < 0.001, compared with controls by a Student *t*-test. **(D–F)** The phosphorylation of JNK **(D)**, P38 **(E)**, and STAT3 **(F)** were up-regulated in HiBECs which were stimulated with PPT and 17β-estradiol, but, it was reduced by fulvestrant (*n* = 3, mean ± SEM). *p < 0.05, **p < 0.01, ***p < 0.001, determined by ANOVA with a Tukey test. **(G,H)** SP600125 (2 μM), SB203580 (2 μM) and a combination of both were added to HiBECs 2 h before stimulation with 17β-estradiol (10nM) and PPT (100 nM). At same time, 17β-estradiol and PPT were added to HiBECs parallel with vehicle. **(G)** Cell culture supernatants of all groups (17β-estradiol (10 nM), SP600125 (2 μM), SB203580 (2 μM) and a combination of both) were collected and analyzed by Elisa for IL-6, IL-8, and TNF-α protein expression in HiBECs. *p < 0.05, **p < 0.01, ***p < 0.001, determined by ANOVA with a Tukey test. **(H)** Cell culture supernatants of all groups (PPT (100 nM), SP600125 (2 μM), SB203580 (2 μM) and a combination of both) were analyzed by Elisa for IL-6, IL-8, and TNF-α protein expression in HiBECs (*n* = 3, mean ± SEM). *p < 0.05, **p < 0.01, ***p < 0.001, determined by ANOVA with a Tukey test. All data are representative of three independent experiments.

As previously reported, estrogen induces cholestasis and cholangiocyte proliferation via activating the MAPK pathways (ERK1/2, JNK, and P38) (23, 24). In addition, P38-MAPK is often over activated within inflamed tissues and accounts for the progression of biliary cirrhosis (25). Hence, we wanted to find out whether ERα-induced pro-inflammatory cytokines expression in HiBECs via activated MAPKs signaling pathways. Firstly, JNK and P38 phosphorylation were tested after ERα was activated. HiBECs were treated with 17β-estradiol (10 nM), and those proteins phosphorylation were detected by immunoblotting analysis. The results indicated that the levels of P-JNK and P-P38 were up-regulated in HiBECs (Figures 5A,C). At the same time, we detected whether the expression of tyrosine-phosphorylated STAT3 (P-STAT3) that is a key protein involved in cytokines expression was also up-regulated in HiBECs. P-STAT3 can be translocated to the nucleus where they regulate transcription by binding to specific DNA sequences (26). In addition, it is reported that P38/STAT3 pathway is involved in inflammatory disorders (27). As expected, our results showed that the levels of P-STAT3 was up-regulated (Figures 5A,C). It was worth noting that the STAT3 activation was blocked by SB203580 (a P38 activation inhibitor), but not affected by SP600125 (JNK activation inhibitor) (Supplementary Figure 4). Hence, we considered JNK and P38/STAT3 accounted for 17β-mediated cytokines expression in HiBECs.

Subsequently, we wanted to confirm that the phosphorylation of JNK, P38, and STAT3 were associated with ERα activation. Hence, as mentioned above, 17β-estradiol (10 nM), and PPT (100 nM) were added to HiBECs parallel with vehicle. And fulvestrant was added to HiBECs 2 h before stimulation with 17β-estradiol. Our results showed that PPT could induce JNK, P38, and STAT3 phosphorylation (Figures 5B,D–F) as well as 17β-estradiol (Figures 5C–F). Furthermore, fulvestrant showed an inhibitory effect on the phosphorylation of JNK, P38 and STAT3, which should be up-regulated by 17β-estradiol (Figures 5B,D–F).

Finally, we wanted to determine the IL-6, IL-8, and TNF-α expression in HiBECs after JNK and P38 pathways were blocked by SP600125 and SB203580.2 μM SP600125, 2 μM SB203580 and a combination of both were added to HiBECs 2 h before stimulation with 17β-estradiol and PPT. And, 17β-estradiol, PPT were added to HiBECs parallel with vehicle. The cell culture supernatants of all groups were collected and analyzed by Elisa kits. We found that the levels of TNF-α were down-regulated in pretreatment of SP600125, SB203580 and a combination of both, when compared with 17β-estradiol and PPT (Figures 5G,H). Although the levels of IL-8 were not affected by pretreatment with SP600125, it was reduced by pretreatment with a combination of both (Figures 5G,H). However, at same time, we did not find significant statistical difference in IL-6 expression in HiBECs that were pretreated with SP600125 or SB203580, when compared with 17β-estradiol and PPT (Figures 5G,H). However, it was down-regulated by pretreatment with SP600125 and SB203580 (Figures 5G,H). Taken together, our results showed that ERα-mediated pro-inflammatory cytokines expression was via activated JNK and P38/STAT3 pathway.

### ERα Activation in HiBECs Facilitates PBMCs Recruitment

**Figure 6:**
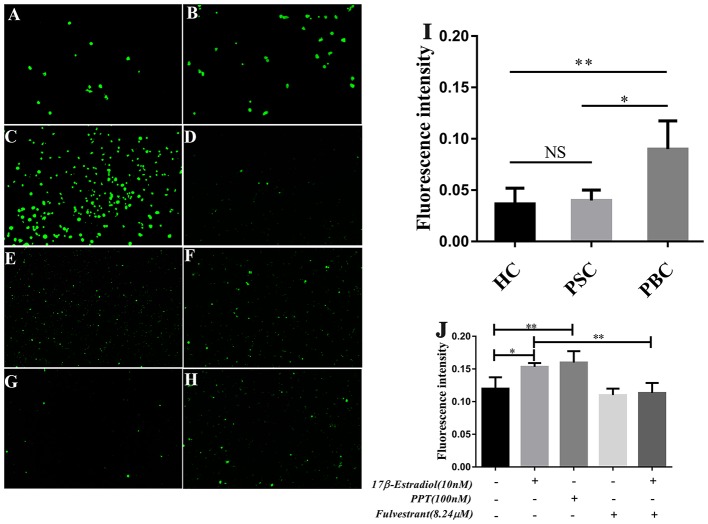
PBMCs activation in PBC patients and prone to binding to HiBECs after ERβ expression was up-regulated. **(A–C)** PBMCs were extracted from 10 mL peripheral blood of PBC patients (*n* = 5), PSC patients (*n* = 3) and HC (*n* = 3).They were stimulated with 10 mg/mL phytohemagglutinin for 72 h. Then, BCECF-AM was added to PBMCs for 1 h and co-cultured with HiBECs. The adhesion rates of PBMCs were reflected by fluorescence intensity) (original magnification, ×200). **(I)** The adhesion rates of PBMCs were higher in PBC patients, compared to PSC and HC, *p < 0.05, **p < 0.01. The *p*-value was determined by Student *t*-test. **(D–H)** PBMCs were extracted from 10 mL peripheral blood of HC (*n* = 3), and they were stimulated with 10 mg/mL phytohemagglutinin for 72 h. Then, BCECF-AM was added to PBMCs for 1 h and co-cultured with HiBECs that were treated by 17β-estradiol (10 nM) **(E)**, PPT (100 nM) **(F)** and fulvestrant (8.24 μM) **(G,H)** (original magnification, ×100). **(J)** The adhesion rates of PBMCs were higher in groups that were treated with 17β-estradiol and PPT, and it was blocked by fulvestrant (8.24 μM). *p < 0.05, **p < 0.01, determined by ANOVA with a Tukey test. All data are representative of three independent experiments.

In our previous assay of immunoblotting, we found the expression of PDC-E2 was up-regulated in HiBECs that were treated with 17β-estradiol (Supplementary Figure 5). PDC-E2 is the immunodominant autoantigen of PBC, and it is involved in breakdown of immunologic tolerance in a genetically susceptible individuals (28). Abnormal expression of PDC-E2 in normal HiBECs may activate immune system. Indeed, in this assay, we found that there were lots of activated immune cells in peripheral blood (PB) of PBC patients, and more prone to bind to HiBECs when compared with HC and patients with primary sclerosing cholangitis (PSC) (Figures 6A–C,I). Landi et al. (29) have proposed that the serum levels of multiple pro-inflammatory cytokines (IL-6, IL-8, IL-2, IFN-γ, IL-23, and IL-1β) in PBC patients were higher than that in PSC and HC, which may be related to immune cells activation. However, as mentioned above, the serum levels of pro-inflammatory cytokines were positively correlated with ERα expression levels in small bile ducts of PBC patients. Hence, we wanted to observe the immunogenicity of HiBECs after ERα activation.

PBMCs were isolated from 10 mL of blood samples of 4 HC. To obtain mitogen-activated immune cells, the PBMCs were stimulated with 10 mg/mL of phytohemagglutinin for 72 h. Then, BCECF-AM was added to PBMCs for 1 h. Meanwhile, 17β-estradiol (10 nM) and PPT (100 nM) were added to HiBECs parallel with vehicle and the fulvestrant was added to HiBECs 2 h before stimulation with 17β-estradiol. The PBMCs were added to HiBECs, which had been cultured for 48 h. The results showed that the fluorescence intensity of 17β-estradiol (10 nM) and PPT (100 nM) groups were significantly higher than that of the controls (0.1513 ± 0.005, 0.1628 ± 0.017 and 0.1196 ± 0.016, respectively) (Figures 6D–F,J). Indeed, we found that the ability of PBMCs binding to HiBECs was reduced by fulvestrant (8.24 μM), when compared with 17β-estradiol (10 nM) groups (Fluorescence intensity: 0.1513 ± 0.005 vs. 0.0698 ± 0.018) (Figures 6G,H,J). In addition, we repeated this assay using primary HiBECs and PBMCs that from same PBC patient and patients with Chronic Hepatitis B (CHB). The results showed that PBMCs were prone to adhere to HiBECs that were isolated in PBC patient than in CHB patients (Supplementary Figure 6). Taken together, ERα activation in HiBECs would attract more PBMCs recruitment, which may be associated with pro- inflammatory cytokines.

### Up-Regulated Expression of CD54 in HiBECs After ERα Activation

**Figure 7:**
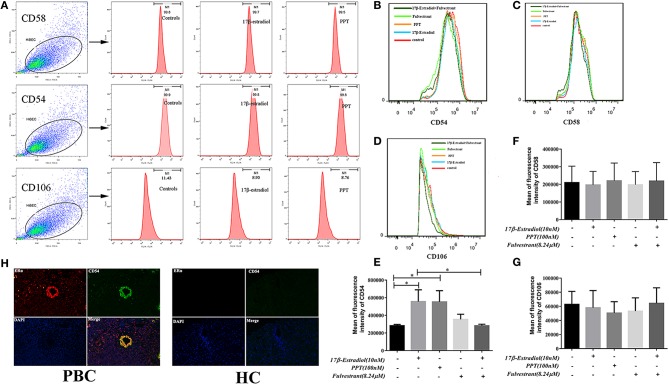
CD54 expression in HiBECs was up-regulated by 17β-estradiol and PPT. **(A)** Gating strategy used after immunostaining to determine HiBECs. **(B–D)** HiBECs are highly expressed CD54 **(B)** and CD58 **(C)**, while there are rarely expressed CD106 **(D)**. **(E–G)** The fluorescence intensity of CD54 was significantly increased in HiBECs that were stimulated with 17β-estradiol (10 nM) and PPT (100 nM) **(E)**, and it was not observed any significant change in CD58 and CD106 expression **(G)**. **(H)** Location of CD54 was concentrated on ERα-positive bile ducts in PBC patients, but not in HC. *p < 0.05, determined by ANOVA with a tukey test. All data are representative of three independent experiments.

Previous studies indicate that the expression of CD54 (ICAM-1), CD58 (LFA-3), and CD106 (VCAM-1) on HiBECs is altered by exposure of proinflammatory cytokines (IFN-γ and TNF-α), and they are functional and necessary for the activity of cytotoxic effector lymphocytes (19). As described above, the pro-inflammatory cytokines might account for PBMCs recruitment. Hence, we determined whether PBMCs were more prone to bind to HiBECs was associated with up-regulated expression of adhesion molecules caused by ERα-mediated pro-inflammatory cytokines. In the present study, flow cytometry was used to determine the expression levels of adhesion molecules (CD54, CD58, and CD106). Our results showed that HiBECs had highly expressed levels of CD54 (99.6% ± 0.21, 99.4% ± 0.38 and 99.5% ± 0.26 for controls, 17β-estradiol and PPT, respectively) (Figures 7A,B) and CD58 (99.0% ± 0.55, 99.2% ± 0.81 and 99.5% ± 0.32 for controls, 17β-estradiol and PPT, respectively)(Figures 7A,C), while they rarely expressed CD106 (11.43% ± 0.24, 8.93% ± 0.10 and 8.76% ± 0.12 for controls, 17β-estradiol and PPT, respectively) (Figures 7A,D). However, 17β-estradiol and PPT can promoted CD54 expression, which was reflected in fluorescence intensity (Figure 7E). In addition, there were no any significant changes in CD58 and CD106 expression in HiBECs treated with 17β-estradiol and PPT (Figures 7F,G). As expected, fulvestrant had a negative role in regulating CD54 expression (Figure 7E).

To further confirm the results from *in vitro* assays, we selected some biopsies from 3 PBC patients, and tested whether CD54 expression can be detected in ERα-positive bile ducts using immunofluorescence. Overall, this results were consistent with flow cytometry, and the location of CD54 was concentrated on ERα-positive bile ducts (Figure 7H). Hence, our results showed that immunogenicity of HiBECs was amplified by ERα-mediated CD54 expression.

### JNK, STAT3 Activation and Cytokines Expressions Are Associated With ERα-Positive Bile Ducts

**Figure 8:**
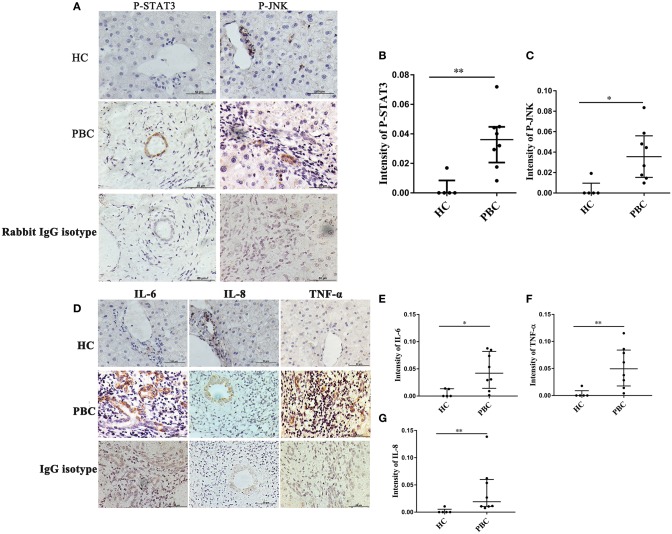
JNK, STAT3 activation and cytokines expression in liver biopsies of PBC patients. **(A)** A significant immunohistochemical JNK and STAT3 positive expression in interlobular bile duct of PBC patients (*n* = 8) (middle panel), but negatively in HC (*n* = 5) (upper right panel) (original magnification, ×400). **(B,C)** JNK, STAT3 activation was higher in PBC patients, *p < 0.05, **p < 0.01 compared with HC by a Mann–Whitney test. Data are represented as median with interquartile range **(D)** The expression of IL-6, IL-8, and TNF-α had tested positive in interlobular bile duct of PBC patients (*n* = 8) (middle panel), but negatively in HC (*n* = 5) (upper panel) (original magnification, ×400). **(E–G)** The expression of IL-6 **(E)**, IL-8 **(F)**, and TNF-α **(G)** were up-regulated in PBC patients, *p < 0.05, **p < 0.01, compared to HC. The *p*-value was determined by a Mann-Whitney test, data are represented as median with interquartile range, *P* < 0.05 was considered significant. PBC, primary biliary cholangitis; HC: healthy control; IL-6, interleukin-6; IL-8, interleukin-8; TNF-α; tumor necrosis factor-alpha; ERα, estrogen receptor alpha.

As indicated above, JNK and P38/STAT3 pathway were involved in ERα- mediated cytokines expression. Hence, we wanted to confirm those results *in vivo*. The liver biopsies were obtained from 8 PBC patients, and those proteins were detected using immunohistochemistry. We found positive staining of P-JNK and P-STAT3 in interlobular bile ducts of PBC patients (Figure 8A), but they were rarely expressed in HC (Figure 8A). At same time, we also found positive expression of IL-6, IL-8 and TNF-α in small bile ducts of PBC patients (Figure 8D). Immunohistochemical analysis showed that those proteins expression levels in small bile ducts of PBC patients were higher than that in HC (Figures 8B,C,E–G).

## Discussion

Intrahepatic bile duct system is one of the target tissues of estrogen. Estrogen plays a vital role in tissue repairing after liver injury, regeneration after liver partial resection, improving insulin resistance and preventing liver fat deposition (30). However, female may develop liver diseases (hepatomegaly, intrahepatic cholestasis and even liver failure), who are receiving long-term estrogen to establish an artificial menstrual cycle (31), pregnant (32), or suffer from CHB (33). The reasons were related with the estrogen levels rise dramatically (33). Hence, estrogen may play a dual role in the physiology and pathology of liver. For a long time, estrogen is using to induce intrahepatic cholestasis of rodents (34). So, estrogen administration is avoided in PBC patients. However, Olsson et al. have proposed that estrogen replacement therapy (as anti-osteoporosis drugs) is safe in PBC patients; they have found that PBC patients with osteoporosis receiving long-time estrogen replacement therapy show no effect on disease development. Even high concentration of estrogen is considered as a protective factor against disease progression in PBC (35). Indeed, some clinical observations of small sample of patients have found that serum enzymes of untreated PBC patients receiving estrogen administration are down-regulated (35–37). Hence, those clinical studies seem to rediscover the concept that estrogen administration in PBC patients exerts deleterious effects on the liver. Nevertheless, as *Invernizzi* reported previously, the levels of AKP and γ-GT were are decreased more than 60% of baseline in PBC patients, who have received tamoxifen (estrogen antagonist), but a complete reversal in case after tamoxifen withdrawal (38). As mentioned above, estrogens exert physiology function mainly via ER receptors. Hence, it is very interesting to clarify the functions of ER receptors in PBC.

ERα, a classical estrogen receptor, is involved in multiple physiological functions of estrogen, such as proliferation of hormone-associated cancer cells and biliary epithelial cells (BECs) (39), and tissue regeneration after partial resection of liver (15, 16). Additionally, it is also shown that estrogen promotes immune cells to product type I IFN via activated ERα (40) and triggers pro-inflammatory cytokines expression in BECs (19). Previously, Alvaro et al. (13) have proposed the ERα expression levels in small bile ducts of PBC patients are correlated with the progression of disease; they consider that the abnormal expression of ERα in ducts is a reason that this disease progresses to ductopenia, although they have not explain the underling mechanism. Recently, Meyer et al. (17) have found that the environment around landfill sites contain higher levels of xenobiotic which activated the human ERα in a dose-dependent manner may trigger PBC. And, this is supported by a follow-up study showing that the soil in the high incidence area of PBC contains a large amount of xenobiotic that can activate ERα (15). Coincidentally, we were considering the reason of the onset of PBC might be associated with the living environment, which stemmed from a survey that screening of anti- mitochondrial antibody subtype M2 (AMA-M2) in 19012 residents of shanghai; pollution and household smoking and living near the landfill sites are considered the risk factors by our analysis (41). In addition, sunset yellow and tartrazine, as Axon A et al. found (42), may induce the onset of PBC. They are very common chemicals in our living environment (coal-derived food and cosmetic colorings) and regarded as novel human ERα activators, which can provoke ERα activation in very small amounts. Hence, the above studies indicate that ERα activation induced by xenoestrogen and estrogen-like compounds of environment plays a pivotal role in the onset of PBC.

However, why post-menopausal women are susceptible to PBC, who have low endogenous estradiol levels is not clear. There are some possible explanations. The first is that the chronic exposure to xenoestrogens or estrogen-like compounds may activate ERα in cholangiocytes, and cholangiocytes will transform into “immunogenicity of individual” (expression pro-inflammatory cytokines and adhesion molecules) after ERα pathway activation. The second is that estrogens stimulate BEC cytokine production is related to ERα-expression, which is higher in female than male BECs (21). The other is that low estrogen levels of post-menopausal women have lost the ability to suppress the immune system, because ERα mRNAs or proteins are expressed in mature immune cells (B cells, CD4^+^T cells, CD8^+^T cells, Monocytes, and DCs), and higher doses of ectopic estrogens typically suppress immune cell activation (43).

In the present study, we proposed that the expression of pro-inflammatory cytokines will be increased in HiBECs after ERα-mediated pathway activation. Multiple studies have confirmed that pro-inflammatory cytokines play an important immune mediating role in the pathogenesis of PBC. IL-6 is a multifunctional cytokine, which is best known for its role in the liver acute phase response. IL-6 can promote hepatocellular carcinoma (HCC) growth and metastasis via antagonism of TNF-α and IL-1β immune response (44, 45). It contributes to the development of autoimmunity disease via activating Th17 cells and inhibiting the Treg cells function. Kumiko et al. (21) have considered that BECs have different IL-6 production, which is the reason that females are more susceptible than males to several biliary tract diseases (PBC, autoimmune hepatitis and polycystic livers). In the course of PBC disease, IL-6 establishes a vicious circle of HiBECs damage. HiBECs will express CD44 after AMA binds to them, and it will induce monocytes recruitment and release IL-6. As a feedback loop, the level of AMA is up-regulated by B cells in the presence of IL-6 (46). Similar to IL-6, serum high levels of IL-8 also contributes to PBC development (47). During the course of chronic liver diseases (CLD), IL-8 mainly activates leukocytes, T lymphocytes and macrophages, and recruits them to the acute inflammatory site (48, 49). The pathogenesis of IL-8 in PBC is related to neutrophil infiltration, and the neutrophilic inflammation in interlobular bile duct will induce a large number of other inflammatory cells migration, and provoke the damage and apoptosis of HiBECs (50). The immune cascade in small bile ducts will be aggravated by lots of immune cells recruitment. Therefore, some researchers have noted that baseline serum levels of pro-inflammatory cytokine (TNF-α) were significantly higher in PBC patients with advanced disease (stage III/IV) than in PBC patients with early disease (51).

However, we found the expression of target antigen (PDC-E2) was also up-regulated in HiBECs, as well as pro-inflammatory cytokines, when ERα was activated by estrogen or PPT. Therefore, we considered that the HiBECs would transform into an “inflammatory cellular phenotype” after ERα was abnormal activated. Hence, there would be a strong attraction for immune cells. As expected, subsequent adhesion experiments found that PBMCs are prone to bind to HiBECs, which were treat with 17β-estradiol and PPT, and it also was proved by primary HiBECs and PBMCs from same PBC patient. In addition, the up-regulated expression of CD54 was to be found in ERα -positive bile ducts and HiBECs that high expression of ERα. CD54 is a ligand for lymphocyte function-associated antigen-1, also known as intracellular adhesion molecule-1 (ICAM-1), is involved in T cell-induced autoimmune disease (52).

Of note, there were some limitations in the present study. First, the patient sample was limited in sample size. It was extremely lack for PBC patients, especially those who needed liver pathology to confirm this disease. Therefore, the positive correlation between ERα expression levels and the levels of cytokines need to be further confirmed by a large sample size. Second, although it was observed that ERα was expressed in the bile duct system, further studies need to detect the ERα expression levels and cytokines at the different PBC stages, providing a more detailed complement on the pathogenicity of ERα in PBC development. Finally, although the number of patients with AMA seemed very large, few people would develop recognizable PBC (53), and it needed to be investigated whether ERα was expressed in small bile ducts of AMA positive patients would be more susceptible to progress to recognizable PBC. In addition, non-response PBC patients who received long-term UDCA therapy would allow us to evaluate the effect of ERα antagonism, which had been exerted a rational therapeutic strategy in sex-based mortality gap in cystic fibrosis and other inflammatory lung illnesses (54). Nevertheless, our results suggested an important role of abnormal ERα expression in cholangiocytes in inducing intrahepatic bile duct inflammation of PBC, providing a therapeutic strategy or a preventive approach for PBC.

## Data Availability Statement

The raw data supporting the conclusions of this article will be made available by the authors, without undue reservation, to any qualified researcher.

## Ethics Statement

The study was approved by the Institutional Review Board of Longhua Hospital (No. 2014LCSY09). All subjects gave written informed consent in accordance with the Declaration of Helsinki.

## Author Contributions

WZ designed and supervised the project and obtained funding. HC, YQ, and BZ performed clinical diagnosis and treatment, collected samples, and contributed to data collection. HC performed bioinformatics and statistical analysis and drafted the manuscript.

### Conflict of Interest

The authors declare that the research was conducted in the absence of any commercial or financial relationships that could be construed as a potential conflict of interest.
