# Supplementary material for: Abnormal Expression of ERα in Cholangiocytes of Patients With Primary Biliary Cholangitis Mediated Intrahepatic Bile Duct Inflammation
Source: Front Immunol. 2019 Dec 5;10:2815. doi: 10.3389/fimmu.2019.02815 (PMC6907097; doi:10.3389/fimmu.2019.02815)
Supplement: Supplementary file 1 [file Data_Sheet_1.pdf]

Supplemental Materials

Table1. Characteristics of PBC patients and Healthy Controls.

	PBC	HC
Age (yrs) Mean±SD	58.71±11.68	56.87±4.58
ALT(U/L) Median(P_{25},P_{75})	28.00(16.00,56.00)	NA
AST(U/L) Median(P_{25},P_{75})	39.00(29.00,68.50)	NA
GGT (U/L)[a] Median(P_{25},P_{75})	131.00(64.00,200.00)	NA
AKP (U/L)[a] Mean±SD	195.29±72.34	NA
TBiL (umol/L) Median(P_{25},P_{75})	13.20(10.15,28.65)	NA
IL-6(pg/mL) Median(P_{25},P_{75})	7.15(6.15,7.67)	5.31(4.81,5.71)
IL-8(pg/mL) Median(P_{25},P_{75})	13.81(12.98,15.06)	10.05(9.37,11.06)
TNF-α(pg/mL) Median(P_{25},P_{75})	6.62(8.54,10.62)	9.84(5.74,7.43)
IL-22(pg/mL) Median(P_{25},P_{75})	35.09(31.93,38.81)	27.28(24.96,29.51)
IFN-γ(pg/mL) Median(P_{25},P_{75})	95.86(87.93,102.31)	77.52(70.82,85.20)
TGF-β(pg/mL) Median(P_{25},P_{75})	536.19(499.67,581.41)	423.16(397.07,472.72)
VEGF(pg/mL) Median(P_{25},P_{75})	38.01(34.84,40.63)	26.98(25.53,28.43)

a, the normal range of alkaline phosphatase is 45-125U/L,g-glutamyl transpeptidase is 10-60U/L in our hospital. NA,not applicable. ALT: Alanine aminotransferase; AST: Aspartate transaminase; γ-GT: G-glutamyl transpeptidase; TBiL: Total bilirubin; IL-6: Interleukin-6; IL-8: Interleukin-8; TNF-α:Tumor necrosis factor-alpha;IL-22:Interleukin-22;IFN-γ:Interferon gamma; TGF-β: Transforming growth factor-beta; VEGF: Vascular endothelial growth factor.

Table 2. List of Primers Used in Real-Time PCR

Gene Name		Sequence	Real-time PCR prodcut size
	Forward	AAAGCAGCAAAGAGGCACTG	137bp
hIL-6	Reverse	TACCTCAAACTCCAAAAGACCAG	
	Forward	GACATACTCCAAACCTTTCCACC	162bp
hIL-8	Reverse	AACTTCTCCCACAACCCTCTGC	
	Forward	CACGCTCTTTCTCTGCCTGCTG	129bp
hTNF-α	Reverse	GGCTTGTCGGGGTTC	
	Forward	CCAGGCTTTGTGGATTTGAC	147bp
hERα	Reverse	GTTCCTGTCCAAGAGCAAGTTAG	
	Forward	TCTCCTTTAGTGGTCCATCGC	181bp
hERβ	Reverse	GAGCATCCCTCTTTGAACCTG	
	Forward	ACCACCATGTACCCTGGACT	103bp
β-actine	Reverse	TTGTTTTCTGCGCAAGTTAGGT	

bp, base pair; h, human

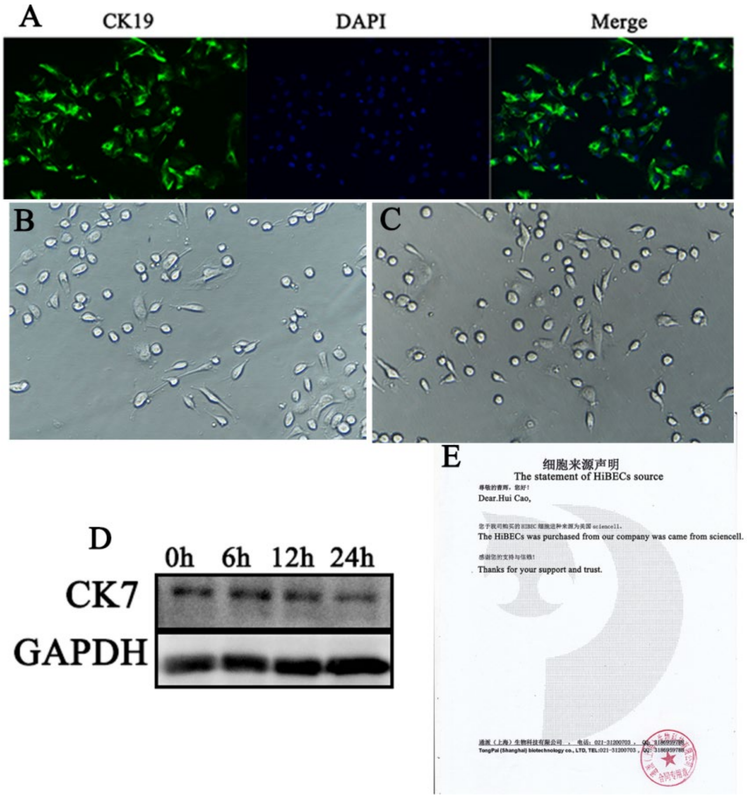

Figure1: **(A-E)**: HiBECs identification. **A**. Immunofluorescence was used to detect the ck19 expression; **B**. Immunoblotting was used to detect the ck7 expression; **B-C**. The morphology of HiBECs was observed by optical microscope; **E**. Certificate was provided by tongpai biotechnology co., LTD.

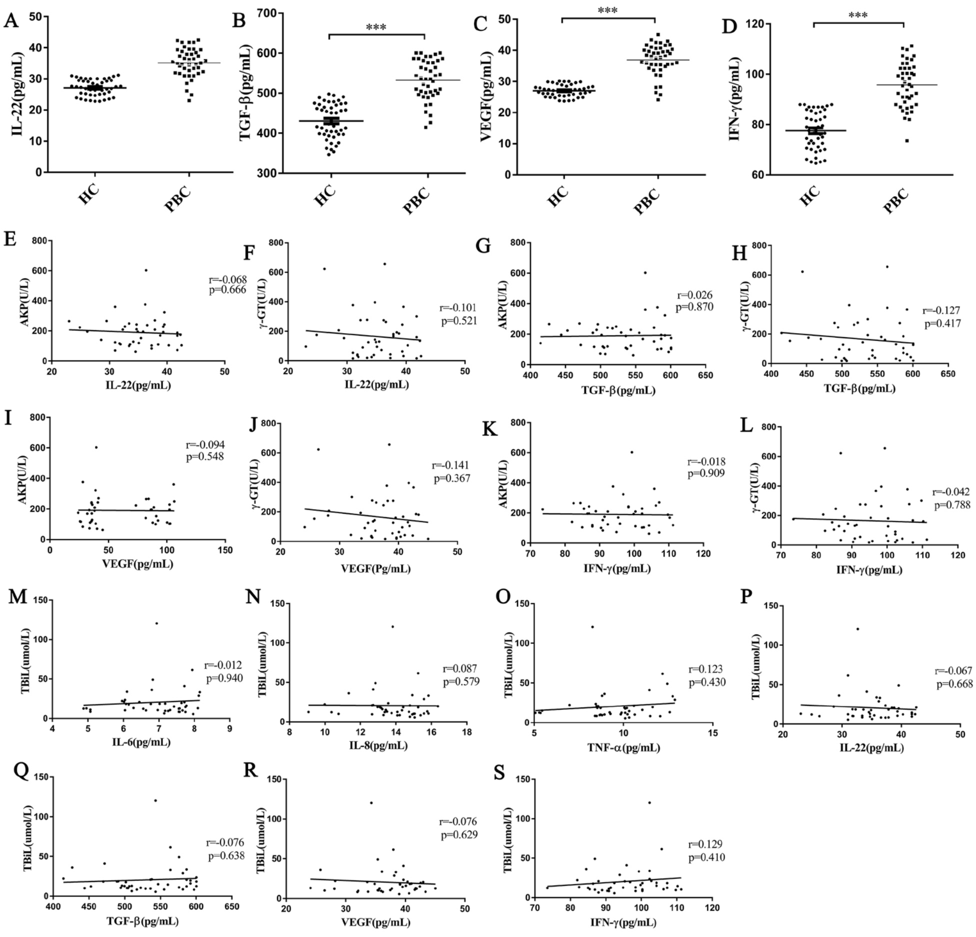

Figure 2: Serum levels of cytokines and correlation with AKP and γ-GT and TBiL. **(A-D)**:Serum levels of IL-22(A), TGF-β(B),VEGF(C) and IFN-γ(D) in PBC patients (n=43) and HC(n=45), ***P <0.001, compared with HC by a Mann-Whitney test. Data are represented as median with interquartile range **(E-L)**: Serum IL-22, TGF-β, VEGF and IFN-γ concentration with the levels of AKP and γ-GT in PBC patients(n=43). **(M-S)**: Serum IL-6, IL-8, TNF-α,IL-22,TGF-β, VEGF and IFN-γ concentration with the levels of TBiL in PBC patients (n=43). The p values were determined by Spearman's rank correlation, P<0.05 was considered significant. PBC, primary biliary cirrhosis; HC: healthy control; IL-6, interleukin-6; IL-8, interleukin-8; TNF-α; tumor necrosis factor-alpha; AKP, Alkaline phosphatase; GGT, g-glutamyl transpeptidase; IL-22: Interleukin-22; IFN-γ: Interferon gamma; TGF-β: Transforming growth factor-beta; VEGF: Vascular endothelial growth factor.

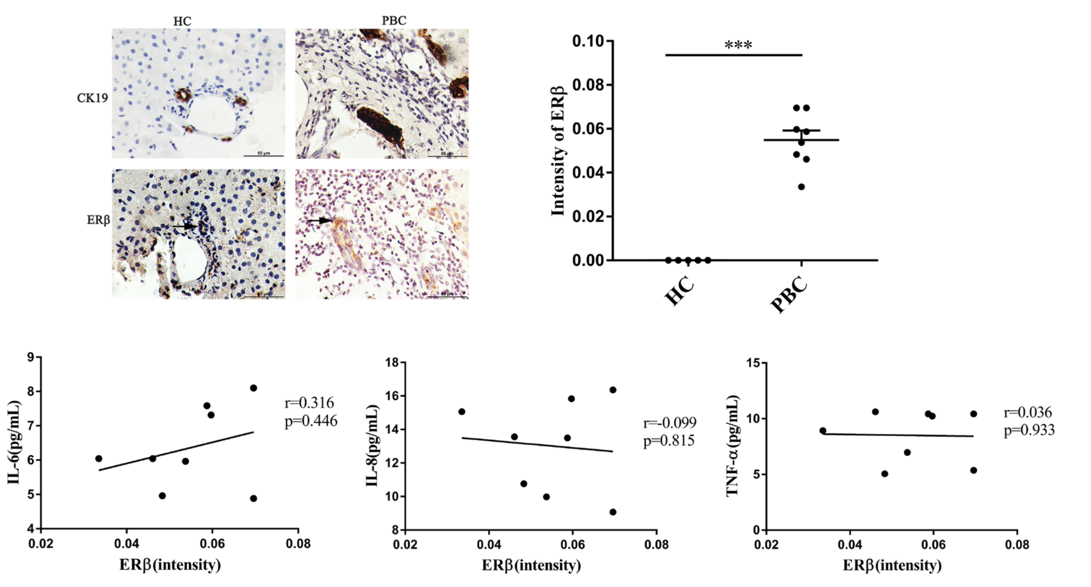

Figure3: ERβ expression in liver biopsies of PBC patients and HC. ERβ expression in small bile duct of PBC patients (upper right panel) were higher than HC (lower right panel)(original magnification, ×400), However,the expression levels of ERβ have not a linear relationship with the levels of cytokines.

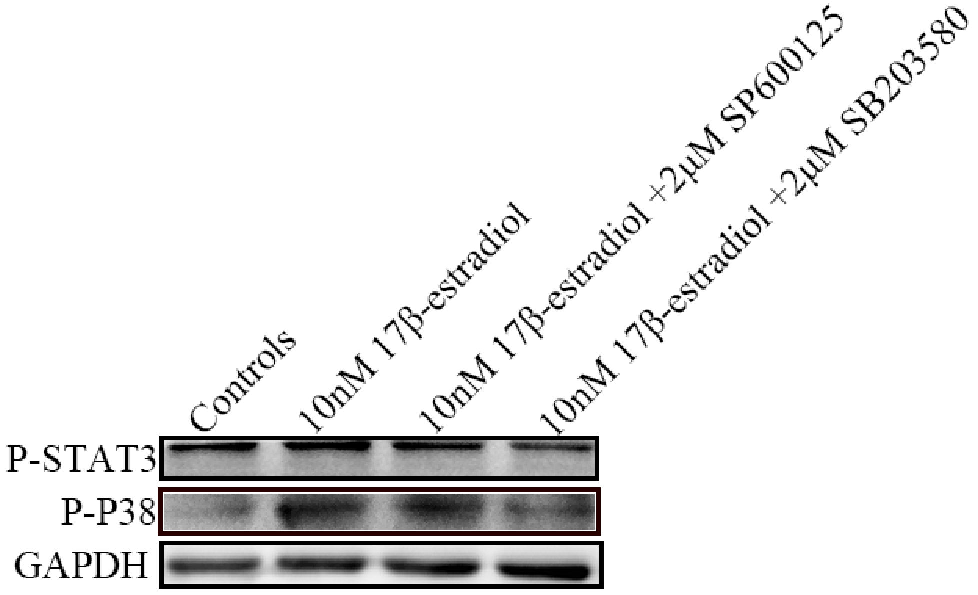

Figure4: *In vitro*, SB203580 can block STAT3 activation, which should be up-regulated in HiBECs by treated with 17β-estradiol.

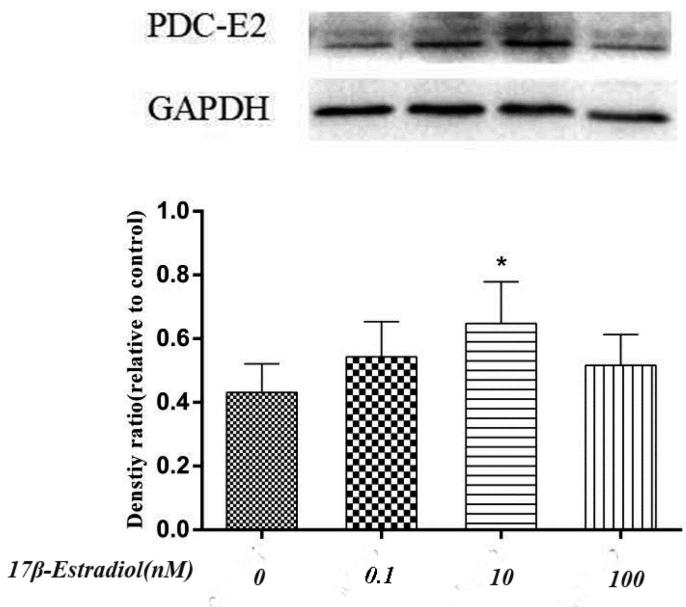

Figure5: *In vitro* 17β-estradiol promoted PDC-E2 expression in HiBECs. HiBECs were treated with 3 concentrations of 17β-estradiol(0.1nM,10nM and 100nM) for 48h. Immunoblotting analysis of PDC-E2 expression in HiBECs. 10nM 17β-estradiol had significantly induced PDC-E2 expression, *p< 0.05 compared with controls by Student t test.

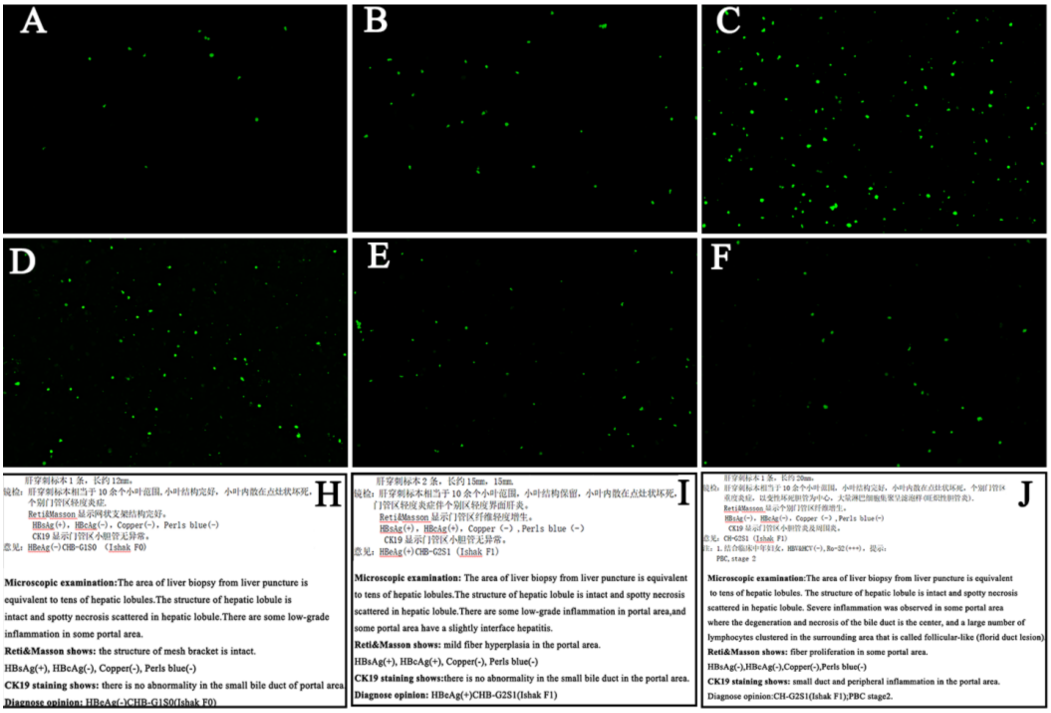

Figure6: PBMCs adhere to HiBECs and the results of liver pathological of three patients. **(A-C):** PBMCs were extracted from 10mL peripheral blood of PBC patients(n=1) and CHB patients (n=2).Meanwhile, HiBECs were also isolated from those patients. PBMCs were stimulated with 10 mg/mL phytohemagglutinin for 72h. Then, BCECF-AM was added to PBMCs for 1h and co-cultured with HiBECs. **A:** PBMCs and HiBECs from CHB patient;**B:** PBMCs and HiBECs from CHB patient 2; **C:**PBMCs and HiBECs from PBC patient. **(D-F)**: Fulvestrant was added to HiBECs which was isolated from PBC patient for 12h **(D)**, 24h**(E)** and 48h**(F)**. **(H-J).**The results of liver pathological of two CHB patients (**A:** patient 1; **B:** patient 2) and PBC patient**(J)**.
